# Book Notices

**Published:** 1869-04

**Authors:** 


### Book Notices.

Treatise on the Diseases of the Ear, including the Anatomy of the Organ. By ANTON VON TROLTSCH, M.D., Professor in the University of Wursburg, Bavaria. Translated and edited by D. B. ST. JOHN ROOSA, M.A., M.D., Clinical Professor of the Diseases of the Eye and Ear in the University of New York, New York; Surgeon to the Brooklyn Eye and Ear Hospital, etc., etc. Second American, from the fourth German Edition. Pp. 565. Price, $4.50. New York: William Wood & Co. 1869.

This is a thoroughly revised and much enlarged edition of an excellent work. The translator has done his work well, and has added many valuable items to the text. There are no diseases concerning which the general mass of practitioners need some additional training, more than those of the ear. They will find in this volume much valuable assistance; and we commend it to their attention.

A Treatise on the Diseases of Infancy and Childhood. By J. LEWIS SMITH, M.D., Curator to the Nursery and Child's Hospital, New York; Physician to the Infants' Hospital, Ward's Island; Professor in Bellevue Hospital Medical College, New York. Philadelphia: Henry C. Lea. 1869.

This is a volume of 620 pages, substantially bound in leather, and printed on fair type and paper. The author has had abundant opportunities for observation and clinical experience, and has embodied the results faithfully in the work before us. We have not had time to examine critically any part of it; but, from a hasty glance at some of the Chapters, we are satisfied that it will form a very valuable addition to the library of every practitioner.

For sale by W. B. Keen & Cooke, 113, 115 State Street, Chicago.

---

Syphilis and Local Contagious Disorders. By BERKELY HILL, M.B., Lond., F.R.C.S., Assistant Surgeon to University College Hospital; Teacher of the Use of Surgical Apparatus in University College, and Surgeon to Out-Patients at the Lock Hospital. Philadelphia: Henry C. Lea. 1869.

This is a small-sized octavo volume of 487 pages, containing an excellent summary of the present knowledge in relation to the nature and treatment of true syphilis and its associate diseases of a more or less contagious character. For the busy practitioner and the student, it will be quite as useful as many of the more voluminous and expensive works.

For sale by W. B. Keen & Cooke, 113 State Street, Chicago.

---

Pennsylvania Hospital Reports. Vol. II. 1869. Philadelphia: Lindsay & Blakiston.

This is an octavo volume of 320 pages, published in excellent style, and filled with matter of direct practical value. It contains twenty-three articles, prepared by Drs. Addinell Hewson, Thomas George Morton, J. M. Da Costa, James N. Hutchinson, Edward Hartshorne, John Ashhurst, William Hunt, J. Forsyth Meigs, John N. Packard, D. Keyes Agnes, George C. Harlon, James Tyson, Joseph G. Richardson, Elliot Richardson, and William Pepper. The materials were furnished mainly by the practice of the hospital, and embrace both medical and surgical topics of permanent interest and value. Price, $5.00.

For sale by Keen & Cooke, 113 State Street, Chicago.

Compendium of Auscultation and Percussion, and of the Physical Diagnosis of Diseases affecting the Lungs and Heart. By AUSTIN FLINT, M.D. Fourth Edition. New York: William Wood & Co. 1869. Pp. 36. Price, $0.50.

---

A History of the Medical Department of the University of Pennsylvania, from its Foundation in 1765, with Sketches of the Lives of deceased Professors. By JOSEPH CARSON, M.D., Professor of Materia Medica and Pharmacy in the University of Pennsylvania, Member of the American Philosophical Society, etc. Philadelphia: Lindsay & Blakiston. 1869.

This is a good-sized octavo volume of 227 pages, well bound in cloth. The title gives an exact idea of the contents of the volume; and the well-known ability of Dr. Carson is sufficient evidence that both the historical account of the University and the biographical sketches are written with fidelity and elegance. Price, $3.00.

---

Quarterly Summary of the Transactions of the College of Physicians of Philadelphia; from December, 1866, to December, 1868, inclusive. Philadelphia: Collins, Printer, 705 Jayne Street. 1869.

Twenty-four pages of this fasciculus of the Transactions of the College of Physicians of Philadelphia, are occupied with abstracts of papers read, and discussions had, before the Society on matters of practical interest; and the remaining twenty-four with interesting biographical sketches of the late Drs. T. E. Beesly, C. W. Pennock, and Francis West.

---

The Part Taken by Nature and Time in the Cure of Diseases. A Dissertation, for which a Prize was awarded to JAMES F. HIBBARD, M.D., by the Massachusetts Medical Society, 1868.

This is a monograph, or, more properly, an essay, of 46 printed pages, in paper cover.

It is written in good style, and we congratulate the author on his success in competing for an honorable award. On the subject discussed in the essay we shall have more to say hereafter.

---

On the Microscope in the Diagnosis and Treatment of Sterility. By J. MARION SIMS, M.D. Reprinted from the *New York Medical Journal*, for January, 1869.

This is a pamphlet of 25 pages, on a subject hitherto obscure and difficult of investigation.

In it, Dr. Sims gives a very interesting account of the manner in which the microscope may enable us to arrive at far more certain conclusions concerning the causes of sterility, than is possible to do without its use. He is entitled to the thanks of the profession for this valuable addition to our methods of investigating and treating human ailments.

---

Operation of Vesico-Vaginal Fistula, without the Aid of Assistants; with a View of the relative Merits of the Clamp, interrupted Silver, and Button Sutures. By NATHAN BOZEMAN, M.D., of New York. Reprinted from the *New York Medical Journal*, for February, 1869.

This is a pamphlet of 26 pages, illustrated with cuts, and containing matter of much interest and value in relation to the treatment of vesico-vaginal fistule.

---

Archives de Physiologie, Normale et Pathologique. Published by M. M. Brown-Séquard, Charcot, Vulpian. No. 2 for March and April, 1869. Illustrated with 4 plates and 17 figures, executed in excellent style. Printed by Victor Masson & Sons, 17 Place of the School of Medicine, Paris.

This number of the Archives fully sustains the reputation of this most valuable publication. We repeat a previous recommendation, that for all who can read the French language, this periodical is worth many times its cost.

---

Venesection as one of the Means for Arresting Unavoidable Hemorrhage. By C. C. F. GAY, M.D., Member of the Surgical Staff of the Buffalo General Hospital, Buffalo, N. Y.

This is a pamphlet of 8 pages, originally published in the *American Journal of Medical Sciences* for the year 1869. The author adduces some cases and excellent reasons to show that venesection may be resorted to in some cases of placenta prævia for the double purpose of arresting hemorrhage and hastening the dilitation of the neck and os uteri.

---

Practical Painter.—We have received No. 1 of Vol. 1, of a small sheet in newspaper form, with the above title, published monthly at 37 Park Row, New York City. Price fifty cents per annum. To painters we think it would be of interest and value.

